# Clinical Diagnostic Accuracy of Onychomycosis: A Multispecialty Comparison Study

**DOI:** 10.1155/2018/2630176

**Published:** 2018-07-03

**Authors:** David G. Li, Jeffrey M. Cohen, Anar Mikailov, Ramone F. Williams, Alvaro C. Laga, Arash Mostaghimi

**Affiliations:** ^1^Department of Dermatology, Brigham and Women's Hospital and Harvard Medical School, Boston, MA, USA; ^2^Tufts University School of Medicine, Boston, MA, USA; ^3^Division of Dermatopathology, Department of Pathology, Brigham and Women's Hospital and Harvard Medical School, Boston, MA, USA

## Abstract

Although onychomycosis can be diagnosed clinically, many guidelines still recommend pathologic confirmation of the diagnosis prior to initiation of systemic treatment. We retrospectively reviewed results from 541 toenail clippings (160 by dermatologists, 198 by podiatrists, and 183 by other provider types) sent to the Brigham and Women's Department of Dermatopathology between January 2000 and December 2013 for confirmatory periodic acid-Schiff (PAS) testing of clinically diagnosed onychomycosis. Of these, 93 (58.1%), 125 (63.1%), and 71 (38.8%) were sent for confirmation of onychomycosis (as opposed to diagnosis of onychodystrophy) by dermatologists, podiatrists, and other provider types, respectively. Confirmatory PAS stains were positive in 70 (75.3%), 101 (80.8%), and 47 (66.2%) of samples ordered by dermatologists, podiatrists, and other providers, respectively. Our study demonstrates that clinical diagnosis of onychomycosis in the appropriate clinical setting is accurate across specialties. Further prospective investigation on the accuracy of clinical diagnosis of onychomycosis may be beneficial.

## 1. Introduction

Onychomycosis is the most common nail disease in adults, affecting approximately 8% of patients presenting to dermatology and general medical offices [1, 2]. While onychomycosis can be diagnosed clinically, some international guidelines still recommend laboratory confirmation prior to initiating systemic therapy [3, 4]. The most commonly utilized confirmatory tests include periodic acid-Schiff (PAS) staining, nail culture, in-office potassium hydroxide (KOH) preparation of nail clippings, or polymerase chain reaction (PCR) [5, 6], each conferring unique strengths and limitations. Of these confirmatory methods, PAS staining has highest sensitivity for detecting onychomycosis, but it is costly [5, 7]. KOH is more affordable but has lower sensitivity; nail cultures are useful but require weeks for results.

Given variations in practice and effectiveness of confirmatory testing, previous research has questioned the role of confirmatory testing for management of onychomycosis [8–11]. Although the rate of onychomycosis in all-comers with onychodystrophy is estimated to be 50%, the rate of onychomycosis in patients who are suspected by clinicians to have onychomycosis (i.e., the post-test probability after physician assessment) is unknown. In this study, we compare the clinical diagnostic accuracy of toenail onychomycosis among dermatologists, podiatrists, and other physicians (e.g., nondermatologist physician including internal medicine physicians, plastic surgeons, and orthopedic surgeons).

## 2. Materials and Methods

We conducted a retrospective cohort study of patients with toenail clippings sent for PAS stain at Brigham and Women's Hospital between January 2000 and December 2013. We reviewed electronic medical records to identify PAS stains that were performed to confirm a clinical diagnosis of onychomycosis. To determine the clinician's intent for performing nail clipping, we manually reviewed electronic medical record corresponding to the clinical visit associated with confirmatory testing order using the following criteria: (1) clinical note strongly suggested a clinical diagnosis of onychomycosis or used a phrase like “consistent with onychomycosis”; (2) physical examination documented onychodystrophy of fungal etiology; or (3) reason for biopsy was listed as “to confirm onychomycosis.”

PAS stains that were done for clinically unclear or diagnostic purposes were excluded using the following criteria: (1) patient note that contained the term “rule out”; (2) listed a differential diagnosis for nail findings; (3) used a modifier like “possible” to indicate considerable diagnostic uncertainty; or (4) if nail removal was performed therapeutically and not solely to obtain a biopsy specimen.

We performed interrater reliability analysis on a subset of 30 cases (Krippendorff's alpha=0.69) before reviewing all cases. Within each provider type, we calculated the proportion of eligible cases that was sent for confirmatory testing, and the proportion that was PAS positive (gold-standard of diagnosis). Statistical significance was calculated using chi-squared tests.

## 3. Results 

**Figure 1:**
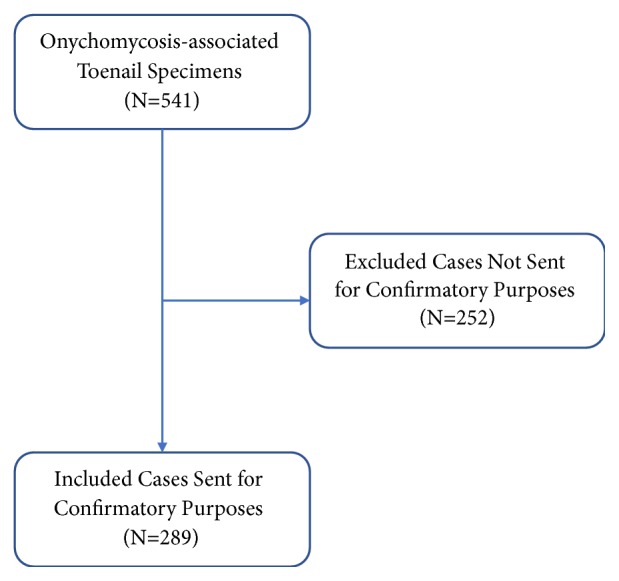
Selection of confirmatory onychomycosis specimens.

We identified information pertaining to 541 onychomycosis-associated toenail specimens sent for PAS stain, of which 160 (29.6%) were sent by dermatologists, 198 (36.6%) by podiatrists, and 183 (33.8%) by other provider types. We excluded 252 onychomycosis-associated cases (46.6%) that were sent for diagnostic rather than confirmatory intent (Figure 1). Of the included cases, 93 (58.1%), 125 (63.1%), and 71 (38.8%) were sent for confirmation of onychomycosis by dermatologists, podiatrists, and other provider types, respectively. Of confirmatory PAS stains, 70 (75.3%), 101 (80.8%), and 47 (66.2%) ordered by dermatologists, podiatrists, and other providers, respectively, were positive (Table 1). Overall, the proportion of correctly diagnosed cases was not statistically different across the three cohorts (p=0.07), with a mean diagnostic accuracy of 75.4%.

## 4. Discussion

In this study, we demonstrated that the clinical diagnosis of onychomycosis exceeds 65% among nondermatologist and nonpodiatrist providers, and it is greater than 75% among dermatologists and podiatrists. Our findings elaborate the test characteristics of clinician physical exam as a function of training status and allow us to compare the value of clinical diagnosis in comparison to expensive and potentially invasive laboratory tests.

Although PAS staining represents a reliable diagnostic tool, its high cost ($138.96) may limit routine use for confirmatory testing within an increasingly cost-conscious healthcare landscape [12]. Additionally, our results show that clinical diagnostic accuracy among dermatologists and podiatrists are approximately comparable to diagnostic accuracy of KOH testing, which confers a sensitivity and specificity of 80% and 72%, respectively [5]. This may be due to a higher prevalence of onychomycosis among patients seen by dermatologists and podiatrists, in addition to more robust clinical expertise pertaining to onychomycosis among these types of clinicians. In settings of high disease prevalence and high clinical suspicion, the incremental benefit of confirmatory testing with either modality may not be worth the increase in cost [8].

These results contribute to an expanding literature debating the utility of confirmatory testing prior to empirical systemic therapy for onychomycosis [8, 9, 13]. Despite the common practice of confirmatory testing prior to initiating therapy, the low cost of systemic treatments such as terbinafine in contrast to the relatively high cost of PAS testing suggests that confirmatory testing may not be cost-effective for management of onychomycosis, although this finding may not be consistent across health care systems and healthcare providers [8, 14, 15]. Our findings also contribute to relevant literature suggesting a high pretest probability of onychomycosis in populations that seek care for nail dystrophy (e.g., dermatology and podiatry clinics) [7, 8].

Although our study contains a moderately large sample size and includes a broad range of provider specialties, our findings must be interpreted in the context of the study design. Our study may be subject to nondifferential misclassification bias attributable to retrospective design. We relied on clinician notes to determine the intent of ordering PAS stains, and it is possible that some cases may have been misclassified. Furthermore, we referenced the result of PAS staining as the gold-standard of diagnosis, and while this test is highly sensitive, there are false positives and false negatives with any laboratory study. Our study is also limited by the single-center design, which may not account for the variations in disease prevalence across different health systems. Finally, our research methodology does not allow us to calculate other statistical measures including sensitivity and specificity.

## 5. Conclusions

These results demonstrate a high clinical diagnostic accuracy for onychomycosis among dermatologists and podiatrists. These findings support reevaluation of the need for standard confirmatory testing prior to onychomycosis treatment, especially in practice settings with a high prevalence of onychomycosis. Further investigation to prospectively measure clinical diagnostic accuracy of onychomycosis among different providers may be beneficial.

## Figures and Tables

**Table 1:** Toenail specimens sent for periodic acid-Schiff (PAS) staining by dermatologists, podiatrists, and other physicians.

	Number of Specimens Sent	Proportion Sent for Confirmation (%)	Proportion PAS Positive (%)‡
Dermatologist	160	58.1	75.3
Podiatrist	198	63.1	80.8
Other Physicians*∗*	183	38.8	66.2

*∗*Other physicians include any nondermatologist physician including internal medicine, plastic surgery, and orthopedic surgery.

‡ P<0.05 comparing cohorts of podiatrists to other physicians. Other comparisons were not statistically significant.

## Data Availability

The data used to support the findings of this study are included within the article.
